# Editorial: Plant-derived natural products: towards novel interventions for neurodegenerative diseases

**DOI:** 10.3389/fnins.2024.1422351

**Published:** 2024-05-07

**Authors:** Natalia P. Alza, Gabriela A. Salvador, Nora E. Gray, Ginpreet Kaur

**Affiliations:** ^1^Instituto de Investigaciones Bioquímicas de Bahía Blanca (INIBIBB) - Consejo Nacional de Investigaciones Científicas y Técnicas (CONICET), Bahía Blanca, Argentina; ^2^Departamento de Química, Universidad Nacional del Sur (UNS), Bahía Blanca, Argentina; ^3^Departamento de Biología, Bioquímica y Farmacia, Universidad Nacional del Sur (UNS), Bahía Blanca, Argentina; ^4^Department of Neurology, Oregon Health and Science University, Portland, OR, United States; ^5^Department of Pharmacology, Shobhaben Pratapbhai Patel School of Pharmacy and Technology Management, Shri Vile Parle Kelavani Mandal's Narsee Monjee Institute of Management Studies (SVKM'S NMIMS), Mumbai, India

**Keywords:** natural products, neurodegenerative diseases, aging, cognitive impairment, motor deficit

Neurodegenerative diseases (ND) comprise heterogeneous disorders characterized by the progressive loss of fundamental abilities, such as thinking, moving, and speaking, thus struggling patients' quality of life. Whereas, clinical phenotypes diverge with behavioral and cognitive changes within different types of ND, there are triggering factors involved in neuronal dysfunction and death in specific brain regions that are common across ND, such as the accumulation of misfolded proteins, the increase in oxidative stress with mitochondrial dysfunction, transition metal accumulation, and neuroinflammation. Treatments for ND remain limited and there are no known cures or disease-modifying therapies for the majority of conditions. Since ancient times, people have used plants as food and medicine, and great advances have been made for understanding therapeutic properties of natural products. Both medicinal plants as well as nutraceuticals represent promising alternatives for the treatment and management of ND. This Research Topic outlines how medicinal plants and nutraceuticals, as well as their derived natural products, can improve brain health and function using several *in vivo* and *in vitro* models that recapitulate ND features.

Regarding the enhancement of cognitive function, researchers shed light on the effect of natural products on memory and learning using Alzheimer's disease (AD) and aging animal models. Abu-Elfotuh et al. focused their work on the potential of probiotics as an add-on therapy with the natural phenol sesamol to counteract neurotoxicity related with an AD-like phenotype. Behavioral tests established a marked improvement in spatial learning and memory performance in Sprague-Dawley rats treated with aluminum chloride as a neurotoxicant with co-administration of both sesamol and *Lactobacillus rhamnosus*. Interestingly, lower levels of amyloid-β and p-tau were observed in the brain of aluminum chloride-intoxicated rats upon the co-treatment. Authors also found that the combined therapy exerted a reduction in brain apoptotic and inflammatory (TNF-α, IL-1β) markers. Speers et al. studied how the administration mode of the aqueous extract of *Centella asiatica* (L.) Urban (CAW) influenced its cognitive-enhancing effects in a model of AD (5xFAD mice). Plasma levels of bioactive constituents were higher when mice received CAW in drinking water rather than diet. Previous works of the same group established that CAW enhances mitochondrial function and decreases oxidative stress in the brain of aging and AD mouse. However, here they demonstrated that CAW impacted little on the expression of neuroinflammatory markers, suggesting that the cognitive benefits of CAW may not be mediated by neuroinflammation modulation.

Cognitive impairment associated with aging is considered a predictor of dementia, and the incidence of certain forms of mild cognitive impairment may be increasing. By using standard behavioral tests to assess different types of memory, Ott et al. examined the effects of Vertigoheel (VH-04), a multicomponent drug (*Ambra grisea, Anamirta cocculus* L., *Conium maculatum*, and *Petroleum rectificatum*), on cognitive performance in aged mice and rats. VH-04 enhanced visual recognition memory, spatial working memory, spatial orientation memory and olfactory memory. Moreover, VH-04 treatment stimulated neurite growth *in vitro* and reversed the age-dependent decrease in hippocampal synaptophysin mRNA expression. Concerning mild cognitive impairment, Shin and Seol argued in favor of linalyl acetate as a potential intervention for this disorder. In this article, authors review numerous *in vivo* and *in vitro* studies indexed in PubMed and Google Scholar databases demonstrating that linalyl acetate could act as an antioxidant and anti-inflammatory agent, and reduce endothelial dysfunction in various experimental models.

The modulation of oxidative stress and neuroinflammation emerges as promising strategies for ND therapies. In Wistar rats challenged with 3-nitropropionic acid (3-NP) to induce Huntington's disease (HD)-like symptoms, Lum et al. demonstrated that the natural glucoxilxanthone mangiferin recovered cognitive function (recognition memory), and motor skills (locomotor activity, neurological scoring, rotarod performance and grip strength). Mangiferin was found to modulate oxidative stress in rat brains, and prevent the rise of pro-inflammatory markers (TNF-α, IL-6, IL-1β) in the hippocampus, striatum and cortex. In addition, massive neuronal degeneration in striatum and cortex regions of rats exposed to 3-NP was reverted by mangiferin. Oxidative stress was also modulated by the aqueous extract of *Mentha rotundifolia* (L.) Huds (85% kaempferol glucuronide) in the work by Boualam et al. The main findings point toward a behavioral enhancement (locomotor activity, motor coordination, anxiety-like behavior, and short-term memory) after *M. rotundifolia* pre-treatment in Wistar rats exposed to H_2_O_2_.

Paraquat (PQ) exposure is widely used as a Parkinson's disease (PD) model, and suitable for behavioral assessment when studying new agents for fighting this ND. Ait Lhaj et al. explored the neuroprotective effect of *Arbutus unedo* L. aqueous extract (AU) in the offspring in which PQ caused neurological deficits. Remarkably, daily post-treatment with AU (enriched in polyphenols) during the 21 days of the rat's pregnancy impacted their progeny by lessening the motor dysfunction and akinesia displayed by PQ exposure in mothers, accompanied with an inhibition of dopamine depletion, and lower iron accumulation in the striatum.

Finally, a systematic review conducted by Moura et al. indicates that there is a noticeable gap in the search of natural products for the treatment of ND. Authors reviewed studies (2002–July 2023) investigating the neuroprotective effects of plant crude extracts, and isolated molecules in models of injuries in the nervous system (traumatic lesions, stroke, and ND). Among 5,521 works, they found 14 suitable for analysis, focusing on effects as neuroprotection against oxidative stress, regulation of the inflammatory response, improvement of tissue alterations, motor and cognitive recovery. Three studies aimed at the exploration of pure natural products: in spinal cord injury models, shogaol recovered hindlimb reflexes and proanthocyanidin enhanced locomotor function; and in a mouse model of multiple sclerosis, periplocoside E reduced the severity and duration of paralysis.

In summary, the articles featured in this Research Topic provide strong basis for natural product-based interventions in ND, evidenced by their ability to improve cognitive function and/or motor skills in various AD, PD, and HD animal models. Studies considered the events occurring in ND, such as oxidative stress, neuroinflammation, and modulation of proteins involved in the pathogenesis (amyloid-β, tau), and how natural products modulate them as the basis of the mechanism of action.

## Author contributions

NA: Writing – original draft, Writing – review & editing. GS: Writing – review & editing. NG: Writing – review & editing. GK: Writing – review & editing.

